# Internet Use and Access, Behavior, Cyberbullying, and Grooming: Results of an Investigative Whole City Survey of Adolescents

**DOI:** 10.2196/ijmr.6231

**Published:** 2017-08-29

**Authors:** Marco Flavio Michele Vismara, Joseph Toaff, Giuliana Pulvirenti, Chiara Settanni, Emma Colao, Serena Marianna Lavano, Riccardo Cemicetti, David Cotugno, Giuseppe Perrotti, Viviana Meschesi, Roberto Montera, Barbara Zepponi, Umberto Rapetto, Rosa Marotta

**Affiliations:** ^1^ Graduate School of Medical Genetics XXVI Cycle Dipartimento di Medicina Sperimentale della Facoltà di Medicina e Odontoiatria Sapienza University Rome Italy; ^2^ Communication Science and Media Consulting Versailles France; ^3^ Dipartimento di Scienze Cognitive, Psicologiche, Pedagogiche e degli Studi Culturali University of Messina Messina Italy; ^4^ Settanni Consulting Group Rome Italy; ^5^ Medical Genetics Unit Mater Domini University Hospital Catanzaro Italy; ^6^ Dipartimento di Scienze della Salute Life Science and Technology Graduate School Magna Graecia University Catanzaro Italy; ^7^ Lega Italiana Diritti dell'Uomo Rome Italy; ^8^ Telecom Italia Group Rome Italy; ^9^ Faculty of Veterinary Medicine Federico II University Naples Italy; ^10^ Department of Obstetrics and Gynecology Magna Graecia University of Catanzaro Catanzaro Italy; ^11^ Department of Obstetrics and Gynecology Campus Bio-Medico University of Rome Rome Italy; ^12^ IMAGO Psychotherapy Clinic Rome Italy; ^13^ HKAO Consulting Rome Italy; ^14^ Dipartimento di Scienze Mediche e Chirurgiche Magna Graecia University Catanzaro Italy; ^15^ Unità Operativa Complessa di Pediatria Universitaria Azienda Ospedaliera “Pugliese-Ciaccio” Catanzaro Italy

**Keywords:** adolescent, Internet, social media, questionnaires, data collection

## Abstract

**Background:**

According to the Digital Agenda for Europe, the way children use the Internet and mobile technologies has changed dramatically in the past years.

**Objective:**

The aims of this study were to: (1) breakdown the modalities of access and use of the Internet by teenagers to assess risks and risky behaviors; and (2) provide scientific data to evaluate and counsel safe use of the Internet and new technologies by teenagers.

**Methods:**

The study was conducted under the program “Strategies for a Better Internet for Children” started in May 2012 by the European Commission. It represents the main result of the project launched by Telecom Italia, “Anche io ho qualcosa da dire” (I too have something to say), thanks to which many contributions were collected and used to develop a survey. The questionnaire was structured in 45 questions, covering three macro areas of interest. It was approved by the Department Board at University of Magna Graecia’s School of Medicine. After authorization from the regional high school authority, it was administered to all 1534 students (aged 13-19 years) in the city of Catanzaro, Italy.

**Results:**

The data was broken down into three main groups: (1) describing education and access to the Internet; (2) methods of use and social networking; and (3) perception and evaluation of risk and risky behaviors. Among noteworthy results in the first group, we can mention that the average age of first contact with information technologies was around 9 years. Moreover, 78.87% (1210/1534) of the interviewed students reported having access to a smartphone or a tablet. Among the results of the second group, we found that the most used social networks were Facebook (85.78%, 1316/1534), YouTube (61.14%, 938/1534), and Google+ (51.56%, 791/1534). About 71.31% (1094/1534) of the interviewed teenagers use their name and surname on social networks, and 40.09% (615/1534) of them knew all their Facebook contacts personally. Among the results of the third group, we found that 7.69% (118/1534) of the interviewed teenagers have uploaded pictures or movies of which they felt ashamed; 27.05% (415/1534) have received invitations from people they met on the Internet to meet in real life; and 8.67% (133/1534) have accepted such invitations.

**Conclusions:**

The results offer a breakdown of the teenagers’ use of the Internet, focusing on how teenagers learn to use and access it while taking into account factors such as parental coaching, schooling, or self-education. It describes how they approach and interact with social networks and how they perceive risks and risky behaviors on the Internet. Information technology must be seen as an instrument and not as a hindrance. For this to happen, parental guidance, schooling, and medical counseling are needed for a sound development of the child in this critical stage.

## Introduction

...long ago, in the old days of the world before the Great War, with whose beginning so many things began whose beginnings, it seems, have not yet ceased...

1

### The Rise of a New Media

The expression “digital natives” has been originally coined by the educator Mark Prensky [2] to highlight the lack of knowledge shown by the average American teacher in understanding and comprehending the needs of students who had grown up in the era of digital information. After 15 years, a full generation of digital natives exist. They can be considered the main actors of a contemporary world characterized by the proliferation and penetration of information technologies into the social fabric. This spread has indeed led to the formation of a new developmental environment somewhat parallel to the long-established ones (scholastic and domestic), which both educators and social and health operators or workers must take into account.

When the so-called “traditional media” (newspaper, radio, television) talk about adolescents and the Internet, they usually tend to focus on what comes through daily reports, such as cyberbullying, sharing of personal and often private information, shaming, stalking, and so on. Beyond that, however, there is a complex reality made of dynamic and carefully maintained social relations in which every subject is, at the same time, creator and user or consumer of contents [3,4]. The Internet was not created with youngsters in mind, but now it is a huge part of their life as they engage in a multitude of Web-based daily activities.

The pervasive nature of this new environment and the astonishing speed with which it has settled among adolescents has increased the natural gap existing between teenagers and their tutors [5].

### The Role of Parents, Tutors, and Health Care Professionals

Parents and social or health operators tend only marginally to take part in these changes due to the often incorrect or poor understanding they have of the Internet and the many activities that adolescents carry out within it [6,7].

Posting contents, pictures, videos, attending online public debates on forums and communities with a simple click and creating customized personal profiles allows the weaving of increasingly wider social networks [8], and they are all elements that, beyond virtually extending the already existing face-to-face interactions managed in more traditional contexts (such as school and playgrounds), also carry out important elements of novelty (see “Discussion” section). These elements can have consequences [9] on the emotional, cognitive, and psychosocial development of children and teenagers that are still largely unexplored. Realizing the depth of the relations built through these new ways of communication represents an unavoidable step toward reaching a full understanding of the sometimes tragic events we already read about on the news [10]. According to several authors [11], youngsters are exposed to a vast array of risks in online that could be grouped in three main categories. The first one include Internet technology risks and refers to content-related (exposure to illegal or potentially harmful contents) and contact-related risks (cyber-grooming, forms of Web-based harassment such as cyberstalking and cyberbullying, exposure to illegal or hateful interactions) that are likely to arise in this particular kind of environment. A second one regards privacy and security risks and may be particularly evident in the use of social networks often carried without adequate understanding of potential short- and long-term consequences (ie, over sharing and sharing of problematic contents). Finally, consumer risks related to Web-based advertising and marketing that may exploit the lack of fully developed critical thinking skills leading to overspending and fraudulent transactions. Other concerns commonly regard abuse of Internet usage by teenagers and fear that it may substitute real-life interactions and therefore be noxious to the social and emotional development of subjects.

Several measures have been proposed to manage such issues and many initiatives from various stakeholders coexist at different and complementary levels [11]. However, a great deal remains to be done in order to better integrate these different efforts and evaluate their effectiveness in making the Internet safer. At the same time, such policies must keep in mind that the Internet represents not only a new and potentially dangerous environment, but also an opportunity as well as a widespread and useful tool in the hand of youngsters.

In order to reach these goals, a better assessment of how youngsters experience, use, and interact on the Internet is required. The primary scope of this study is, therefore, to define levels of involvement, levels of awareness of potential dangers and misconducts, and conditions of social network platforms and Internet usage displayed by a wide sample of adolescents. A deeper knowledge of the phenomenon based on a rich qualitative database is a primary requirement for any further interpretation and action related to the use of these kinds of technologies by adolescents. Moreover, since the classification of natural objects in science is unstable [12], we think that providing such qualitative data could be useful for future comparative studies and required to later evaluate the evolution and development of the phenomena.

## Methods

### Distribution and Coverage of the Survey

We conducted this study under the program “Strategy for a Better Internet for Children” [13] started in May 2012 by the European Commission. Its main goal is to create a database of information regarding Internet usage and behavior of adolescents on the Internet. Such data may be further used to identify a practical strategy to make the Internet a safer place for youngsters, and give them and their tutors the right tools and recommendations to live in this new environment without risks.

In order to achieve the goal, we developed a survey based on a rich preliminary exploratory groundwork, in collaboration with the European Union (EU) Commission, telecommunication corporation (Telecom Italia), and local educational authorities. The aim was to quantify the tendency of youngsters to adopt potentially risky actions on the Internet [14-17] and roughly map their behavior to gather useful data for future epidemiological, behavioral, social, and health-related researches on the issue. As suggested by the program “European Strategy for a Better Internet for Children” [13], it is important to collect data about Internet usage by adolescents in order to help them acquire the skills and tools they need to fully and safely benefit from using the Internet as well as unlocking a potential market for interactive, creative, and educational Web-based content. The data also represents a starting point of investigation to describe the effects that Web technologies have on the psychosocial and cognitive development of children and teens.

On June 3, 2013, broadcasting and telecommunication corporations were invited by the EU Commission to actively contribute and take part in the project. On June 26, 2013, Telecom Italia promptly gave life to the “Stati Generali della Tutela dei Minori” (Estates General for minors’ protection) and arranged a meeting, well-advertised and promoted by social media, which was attended by institutions, associations, and specialists of the field [18].

The main result of this meeting was the launch of “Anche io ho qualcosa da dire” (I too have something to say), a project directed by Umberto Rapetto. After a census of potential contributions and experts, we edited a syllabus, consisting of a list of key themes that were worth studying in relation to our main purpose. The chosen topics were then examined and debated during a series of events specifically conceived to captivate and involve the social fabric of some Italian regional centers with the intention of gathering ideas, point of views, and suggestions as much as possible. Through this exploratory work, we engaged with the public and identified some key themes that informed our survey questions.

The initiative was launched in Genoa on October 14, 2013 at the high school “Istituto Magistrale Pertini,” and it later reached the cities of Bari, Catanzaro, and Trento. It consisted in engaging the community of each city for 5 days with information campaigns, cultural spaces, and meetings dedicated to explore this issue. The next step was collecting the most significative contributions to draft, on the base of this experience, a pool of questions that underwent respondent validation. The questionnaire was then approved by the Board Commission of the Department of Medical Sciences of Magna Graecia University of Catanzaro and by the local educational authorities, which entitled its administration to all the students of public high schools of the regional center of Catanzaro. This study, therefore, consists of a descriptive statistical analysis, a census about Internet usage habits of all the teenagers (aged 14-18 years) that attended public high schools of an important Italian regional center, the city of Catanzaro, during school year 2013-2014.

### The Questionnaire

We specifically drafted the questionnaire to investigate the frequency of access and Internet usage of the adolescent population of the city, which was the main target of our study. We structured the survey in three macro thematic areas: (1) learning and network access, (2) conditions of use and social networking, and (3) risk perception and behaviors. In the first one, we conducted a preliminary analysis on the Internet usage habits of the teenagers and the ways through which they came to acquire the practical competences required to surf the Internet. The focus of the second cluster was directed on the usage of social networking platforms and the managing of their personal identity on the Internet, whereas the last one analyze the adoption of potentially dangerous behaviors and their perception by the study subjects.

The overall questionnaire consists of 45 close-ended questions (available in Multimedia Appendix 1) covering the three macro areas of interest above mentioned. Each question was further designed to be clear, short, and precise to be easily understood and reduce fatigue among respondents. The answers were collected anonymously to minimize selective suppression of information by the subjects due to possible feelings of shame or social acceptance. The majority of questions were conceived to have a yes or no answer in order to simplify the analysis of responses; however, in many cases, we have decided to set more fixed options and allow the possibility to give multiple answers to avoid losing precious information and break any possible yes-or-no pattern effect among the subjects of the survey.

## Results

### Overview

The teaching staff delivered the survey over the academic year 2013-2014 to all secondary school students (aged 14-18 years) in the city of Catanzaro, except a single class (22 subjects). Both tutors and students have shown a great level of collaboration. A total number of 1534 students participated in the study (54.6% males; 43.3% females; 2.2% stated no gender). The cards were then collected and the response rates to each question (Yes or No or white) have been analyzed, divided by gender (Male or Female), and recorded in a data set (available in Multimedia Appendix 1). Results are reported according to the three macro areas, or clusters, described in the Methods section.

### First Cluster: Access and Learning

This cluster aims to explore teenagers’ Internet access routines: type, availability, and ownership of the devices with which the subjects access the Internet. It also aims to investigate from whom the teenagers get their first instructions on how to use the Internet, how they respond to the instructions given, as well as identifying the level of confidence shown to adults in terms of discussing their own activities on the Internet (Table 1).

Of the participants, 79.20% (1215/1534) possess a smartphone and/or a tablet for accessing the Internet. This fact agrees with several international research projects, according to which teenagers are becoming “cell-mostly,” or even “cell-only” users, with the implementation (increasingly widespread) of devices such as smartphones or tablets. Conversely, a mere 20.73% (318/1534) accesses the Internet exclusively from their home PC.

We found that 96.87% (1486/1534) of teenagers in Catanzaro own or have access to a computer with an Internet connection. Among them, 72.68% (1115/1534) access the Internet by using their own PC, which is not shared with the rest of the family (Tables 2 and 3).

In 2013, in Catanzaro, teenagers started to use the Internet on average at the age of 9. We found that 53.0% (444/837) of the interviewed males and 41.0% (272/664) of the females were self-taught in the use of the Internet. For 29.85% (458/1534) of the interviewed subjects, Internet use had been taught at home (by parents or relatives) rather than in school. The most commonly given parental advice is about avoiding communication with strangers (81.02%, 1243/1534) and not spreading personal data (75.74%, 1162/1534).

**Table 1:** Internet access.

Question		Total yes	% yes	Male, % yes	Female, % yes	Gender not declared, % yes	Void forms
Do you own a computer?		1115	72.7	40.7	30.4	1.4	0.2
Does your PC have antivirus software?		1377	89.8	49.4	37.8	1.8	0.8
Do you have an Internet connection?		1486	96.9	52.6	42.2	2.0	0.1
Is your Internet connection fast enough?		1275	83.1	45.4	35.4	1.8	0.5
Do you own a tablet or mobile phone?		1215	79.2	44.0	33.2	1.6	0.3
Do you use a tablet or a mobile phone to go online?		1226	79.9	43.7	33.9	1.6	0.7
**Do you use someone else’s device, PC, tablet, mobile phone to go online?**							
	Parents	263	17.1	7.9	8.3	0.4	0.5
	Friends	278	18.1	8.3	8.7	0.5	0.5
	No	1021	66.6	37.7	27.1	1.2	0.5

**Table 2:** Learning.

Question		Total yes	% yes	Male, % yes	Female, % yes	Gender not declared, % yes	Void forms
Has someone taught you something about Internet use?		812	52.9	25.6	25.4	1.3	0.7
**Who taught you to surf the Web?**							
	Parents	484	31.6	15.2	15.3	0.7	0.4
	Teachers	63	4.1	1.7	2.0	0.1	0.4
	Friends	280	18.3	9.3	8.2	0.4	0.4
	Family	433	28.2	14.7	12.7	0.5	0.4
	Myself	463	30.2	18.9	10.4	0.5	0.4

**Table 3:** Tutoring.

Question		Total yes	% yes	Male, % yes	Female, % yes	Gender not declared, % yes	Void forms
**Do your parents surf the Web with you?**							
	Often	142	9.3	4.2	4.6	0.3	0.1
	Sometimes	718	46.8	24.4	21.2	1.0	0.1
	Never	675	44.0	25.7	17.5	0.7	0.1
**Did your parents install filters to limit your Web surfing?**							
	Yes	112	7.3	3.4	3.3	0.4	0.3
	No	962	62.7	35.8	25.5	1.1	0.3
	Don’t know	463	30.2	14.8	14.4	0.7	0.3
Do you think that the practical experience of an adult would help you use the Internet?		1048	68.3	34.3	31.6	1.6	0.8
**Did parents, teachers, or friends make suggestions about how you should use the Internet? If yes, what kinds of suggestions did they make?**							
	They told me never to give my personal data	1162	75.7	38.4	34.6	1.4	1.4
	They gave me a maximum time to stay online	365	23.8	11.6	10.2	0.4	1.6
	They told me not to communicate with strangers	1243	81.0	40.2	38.3	1.5	1.1
	They told me which website I can connect to and what I should do	669	43.6	21.4	19.8	0.8	1.6
	They told me to not send or post pictures that are of me or my family	635	41.4	21.4	17.7	0.8	1.5
	They told me how to behave in case of complications, awkwardness, or fear	923	60.2	29.4	27.8	1.3	1.6
	They explained to me how to use chats and instant messaging	547	35.7	17.6	15.6	0.8	1.6
	They advised me to not use the Internet by myself	234	15.3	6.1	7.1	0.3	1.8
	They advised me to tell them what I do online	523	34.1	15.3	16.5	0.6	1.7
Have you ever told to your parents/teachers/friends what you have seen or done online?		937	61.1	28.5	30.3	1.2	1.0

**Table 4:** Conditions of use.

Question		Total yes	% yes	Male, % yes	Female, % yes	Gender not declared, % yes	Void forms
**Every day, how much time do you spend on the Internet?**							
	1 h or less	624	40.7	22.4	16.8	1.0	0.5
	2-3 h	599	39.0	21.7	15.9	0.9	0.5
	4-6 h	178	11.6	4.9	6.1	0.1	0.5
	>6 h	144	9.4	4.7	4.0	0.1	0.5
**Why do you connect to the Internet? You can give more than an answer.**							
	Do school homework	1225	79.9	41.6	36.2	1.6	0.5
	Get sport, music and info news	1216	79.3	44.7	32.8	1.4	0.4
	Play online	972	63.4	38.0	23.5	1.4	0.5
	Music or video streaming or downloading	1359	88.6	47.5	38.9	1.6	0.6
	Chat and instant messaging	1326	86.4	46.7	37.5	1.6	0.6
	Email	504	32.9	19.4	12.1	0.6	0.7
**Do you have any social network accounts? If yes, which?**		1104	72.0	36.8	33.4	1.4	0.3
	Facebook	1316	85.8	47.7	35.7	1.8	0.7
	Twitter	547	35.7	19.4	13.0	0.6	2.7
	MySpace	180	11.7	5.7	2.4	0.3	3.4
	Google+	791	51.6	29.4	18.5	0.9	2.7
	Pinterest	96	6.3	1.9	0.7	0.1	3.5
	Flickr	118	7.7	2.9	1.3	0.1	3.4
	YouTube	938	61.1	35.9	21.6	1.3	2.3
	Instagram	606	39.5	17.7	18.5	0.7	2.7
	Others	388	25.3	11.4	10.0	0.5	3.4
Do you use a webcam?		865	56.4	28.9	25.6	1.4	0.6
**When do you use a webcam? You can give more than one answer.**							
	Video chat with schoolmates and friends	867	56.5	28.2	26.1	1.4	0.7
	Video chat with friends met online	179	11.7	7.2	3.5	0.3	0.7
	To learn more about the people you met online	56	3.7	2.2	0.7	0.1	0.7
	Take pictures or make videos to post publicly online	230	15.0	7.0	6.8	0.5	0.7

### Second Cluster: Conditions of Use and Social Networking

It consists of two areas of interest. First, we wanted to investigate the teenagers’ social customs, their access to the most common social platforms, the average length of their Internet sessions, and the general purpose of their activity. The second part of the cluster focuses on the use of identities and pseudonyms while on the Internet, and on the usage of devices such as webcams (Tables 4 and 5).

Responses from this second cluster indicate that the majority of the teenagers in Catanzaro use the Internet to download music and movies (88.59%, 1359/1534), to chat and send messages (86.44%, 1326/1534), to search for news (79.26%, 1216/1534), or information for school projects (79.85%, 1225/1534). The most used social networks are Facebook (85.78%, 1316/1534), YouTube (61.14%, 938/1534), and Google+ (51.56%, 791/1534). Only 11.73% (180/1534) of the interviewed use MySpace, which dominated in this age group before the beginning of the “Web 2.0” era.

Of the teenagers in Catanzaro, 71.31% (1094/1534) use their real first and last name on social networks, the remaining 28.68% (440/1534) use pseudonyms, and 12.45% (191/1534) admit to using fake profiles. We find that 40.09% (615/1534) of the interviewed subjects know all of their Facebook contacts personally.

**Table 5:** Social networking.

Question		Total yes	% yes	Male, % yes	Female, % yes	Gender not declared, % yes	Void forms
When you are online, do you use a nickname or a fake identity?		440	28.7	16.9	9.6	0.5	1.6
Do you prefer to use a fake profile on the Internet?		191	12.5	6.2	4.5	0.2	1.6
When you are online, do you feel stronger than in real life?		210	13.7	5.9	5.9	0.3	1.6
Do you lie regularly when you are online?		197	12.8	7.9	3.2	0.2	1.6
**Where do you have more friends?**							
	In real life	1136	74.1	40.4	30.1	1.6	2.0
	On the Internet	384	25.0	10.9	11.9	0.3	2.0
**How many friends do you have on Facebook or other social networks?**							
	<50	103	6.7	3.0	2.0	0.3	1.4
	50-200	315	20.5	10.2	8.3	0.6	1.4
	200-500	438	28.6	14.8	11.7	0.6	1.4
	500-1000	321	20.9	11.3	7.9	0.3	1.4
	>1000	252	16.4	8.3	6.5	0.2	1.4
**How many social network friends do you know in real life?**							
	None	45	2.9	1.1	0.5	0.1	1.3
	A few	54	3.5	1.1	0.8	0.3	1.3
	20-50	100	6.5	2.5	2.6	0.1	1.3
	50-100	178	11.6	5.1	5.1	0.1	1.3
	>100	480	31.3	16.6	12.6	0.7	1.3
	Everyone	615	40.1	22.4	15.6	0.8	1.3

### Third Cluster: Risk Perception and Behaviors

We divided this cluster into three main areas. The first one focuses on Internet use for potentially dangerous behavior, such as bullying and shaming or requests to meet in person somebody known on the Internet. The second area analyzes publishing of compromising photos or videos, and how such photos or videos are exchanged in order to get favors or currency (often used in grooming). The section ends with a series of questions on risk perception about potentially illegal behaviors (Table 6).

We find that 27.05% (415/1534) of teenagers in Catanzaro admitted to having received requests from someone known to them on the Internet to meet in person, with a marginal gap between males and females (24.3%, 204/837 vs 26.0%, 173/664). Only 8.67% (133/1534) of the interviewed subjects have accepted invitations to meet up with strangers. Another potentially dangerous behavior, exchanging photos or videos for PostePay (an Italian debit card that can be recharged on the Internet) or cell phone credit top-ups or other presents, has been found in less than 10% (9.51%, 146/1534) of the cases (Table 7).

Nearly 62.12% (953/1534) of the interviewed subjects admit to uploading photos and videos online. Among them 40.35% (619/1534) report having uploaded more than 50 pictures of themselves, but less than 10% (7.69%, 118/1534) admit to having posted compromising photos or materials they might be ashamed of (Table 8).

The interviewed teenagers are mostly aware of the risks involved with this behavior: 84.35% (1294/1534) know that such behavior can bring legal consequences, and 77.90% (1195/1534) know that the police can gather personal information to investigate illegal behavior.

**Table 6:** Risk perception.

Question	Total yes	% yes	Male, % yes	Female, % yes	Gender not declared, % yes	Void forms
Have you ever been in trouble while you were online?	352	22.9	12.0	8.7	0.6	1.7
Have you ever used the Internet for revenge, to tease or be cruel to someone?	235	15.3	8.0	5.5	0.2	1.6
Are you afraid that someone can disparage you online?	537	35.0	15.6	17.1	0.6	1.7
When you chat, do you talk of things you find embarrassing in real life?	363	23.7	10.2	10.6	0.6	2.3
Have you ever received invitations to meet with people met online?	415	27.1	13.3	11.3	0.5	2.0
Have you ever accepted an invitation to meet with people you met online?	133	8.7	4.8	1.8	0.2	1.8

**Table 7:** Management of online self-image.

Question		Total yes	% yes	Male, % yes	Female, % yes	Gender not declared, % yes	Void forms
Do you post pictures or videos online?		953	62.1	31.5	27.3	1.4	1.9
**How many pictures do you have on your profile?**							
	None	172	11.2	5.5	4.2	0.2	1.4
	1	73	4.8	2.3	1.0	0.1	1.4
	<20	294	19.2	12.8	4.6	0.3	1.4
	20-50	345	22.5	12.1	8.5	0.5	1.4
	>50	619	40.4	17.5	20.7	0.8	1.4
Have you ever sent or posted anything you feel embarrassed about?		118	7.7	4.3	1.4	0.1	1.8
Would you trade pictures of yourself for mobile credit or other things?		146	9.5	5.5	1.6	0.4	2.0

**Table 8:** Risky behavior and awareness.

Question	Total yes	% yes	Male, % yes	Female, % yes	Gender not declared, % yes	Void forms
Have you ever argued with someone online?	658	42.9	22.4	17.8	1.0	1.8
Do you like watching violent material on the net?	248	16.2	12.2	1.4	0.5	2.0
Do you know that you can be reported for your online behavior?	1294	84.4	43.9	36.9	1.8	1.7
Do you think that police could bust you if you do something wrong online?	1195	77.9	40.2	34.1	1.6	2.0

## Discussion

### Principal Findings

A child’s development does not run on completely fixed paths, but rather on possible and differentiated routes. Developmental trajectories are often irregular and unpredictable, and they heavily depend on the effect of environmental and sociocultural factors with which the subject interacts and relates to [8]. Adolescence is a transitory stage of life between childhood and adulthood, characterized by multiple shifts and adjustments, which are part of the acquisition of critical cognitive and social skills. Nowadays, we are witnessing a dramatic change in environmental conditions that are used to house—and, in a sense, drive—this important step, mainly due to the exponentially accelerating technological progress, a real hallmark of our time.

Such changes have several consequences. These concern both the development of the emotional, social, and cognitive skills of children, which may be shifted toward different paths than those with which we are familiar, thereby exposing children to certain risks and dangers. Parental figures and tutors who normally play a major role in child development have only partial knowledge of the new digital media- and Internet-related technologies. This reflects the poor understanding they have of the phenomenon and its dynamics [2], as well as the limited control they have over it. Research specifically oriented to assess the behavior of adolescents on the Internet and the risks associated with such activities seem particularly urgent. Most of the data collected in the past about Internet usage concerns mainly adults and young adults (college students) [19-21]. Only very recently, the focus has shifted toward younger population groups [22-26].

The presented research aims first and foremost to serve as an important fact-finding and investigative tool that could help fill the generational gap that has arisen as a result of the advent of the new digital technologies. We propose a descriptive and broadly representative study of the habits, motives, and styles of Internet access and social networking of adolescents. We believe that it could constitute an excellent database and a starting point for the development of intervention and risk prevention strategies. Some recent studies also specifically want to provide data on adolescent Internet access as well as give useful insight on the main consequences of new media [27,28]. However, because the majority of them are cross-sectional, our research differs in the methodology since we collected data from the entire teenage population of an Italian regional center.

Among the risks associated with Internet use, the growing phenomenon of cyberbullying has a great relevance [29-31]. This term refers to threatening or humiliating activities against others, as well as public shaming mechanisms, which occur through electronic devices [29]. The spread of this and other hazardous practices appears related to some characteristic attributes of communication mediated by new digital technologies: anonymity, synchronization, and accessibility [32]. Anonymity can be total [33] or it can be partial, involving just some aspects (ie, audio and/or visual). Thanks to anonymity the subject has greater control over the quality and quantity of personal information he or she wants to share publicly on the Internet. This can have both positive and negative effects on the development of adolescents. It can lead the subject to think that, once partially or not at all identifiable, he or she can act irresponsibly, impulsively, and, in extreme cases, in a way that can be harmful to himself or herself and to others without bearing responsibility. However, according to data obtained from our survey’s second cluster results, total anonymity is less and less common among young Internet users: 71.31% (1094/1534) of teenagers in Catanzaro use their real first and last name on social networks, 28.68% (440/1534) use pseudonyms, and only 12.45% (191/1534) admits to using fake profiles.

On the other hand, anonymity may also have a positive impact, encouraging self-disclosure leading to greater self-esteem and higher levels of socialization due to lower level of concern about one’s own physical appearance and judgment of peers [33,34]. A certain extent of anonymity can thus lead to higher self-exploration (or self-consciousness), particularly in the sexual sphere, which often suffers censorship or reproach in “real life” due to local cultural and religious habits and traditions. Our data reveal that 23.66% (363/1534) of adolescents in Catanzaro discuss online matters that they would otherwise be ashamed to talk about in person.

The widespread availability of current digital technologies results in higher accessibility for users, especially adolescents, who can be considered the main users of new social medias, and who have the opportunity to choose their social audience. They can stay in touch with people with similar interests, or with people whom they would never be able to meet in real life (ie, due to distance), or they can be introduced to different cultural practices. A greater level of Internet access obviously extends not only the number of opportunities, but also the number of risks. For instance, although traditional forms of bullying are limited in time and space, Internet-related ones do not have these constraints. This specific feature could potentially amplify the magnitude and persistence of the offence [31]. Indeed, 35.00% (537/1534) were afraid that someone could disparage them on the Internet. On the other hand, 15.31% (235/1534) of our teens admitted to having used the Internet for revenge, to tease, or be cruel. Asynchronicity is another characteristic of online communication, and it allows adolescents to have greater control over the information they decide to share due to the possibility of thinking and editing messages before sending them. It can be particularly beneficial for kids who are shy, emotional, or easily embarrassed in offline interactions [35].

Overall then, these opportunities can enrich and ease the identity formation process, but they can also make the subject vulnerable to the cyber-grooming phenomena, such as sexual harassment or unwanted solicitations from strangers [36]. We tried to assess risk perception and hazardous behavior on the Internet in the third cluster of the survey.

Data reveals that 22.94% (352/1534) of the interviewed adolescents claim to have had unpleasant experiences on the Internet, whereas 27.05% (415/1534) have received invitations to meet someone they got to know on the Internet in real life. Our data shows that teens are at least partially aware of the risks associated with such activities: only 8.67% (133/1534) of them have accepted these kinds of meetings. Although a high percentage of the adolescents surveyed admit to uploading photos and videos on the net (62.12%, 953/1534), less than 10%, 9.51% (146/1534) report a willingness to exchange photos or videos with phone cards or gifts. Only 7.69% (118/1534) of respondents say that they have published compromising or embarrassing material on the Internet.

Another main concern associated with Internet usage by adolescents is related to its abuse and compulsive usage. Results from first and second clusters regarding level of accessibility and conditions of Internet usage confirm that our teenagers become familiar with these technologies early in life, at around 9 years of age, and they seem to use them extensively. Of those we surveyed, 96.87% (1486/1534) have access to a computer and an Internet connection, and 79.20% (1215/1534) have a smartphone or a tablet. We found that 43.54% (668/1534) of respondents would rather have an up-to-date smart phone than a scooter, although for the majority (88.65%, 1360/1534: 47.06%, 722/1534 male and 38.39%, 589/1534 female), a day at the seaside remains a preferable option to chatting or playing videogames on the Internet. It therefore seems that Web-based reality is still far from being a substitute for the “real” one.

The learning process involved in properly using new digital devices is increasingly autonomous: although 52.93% (812/1534) of teens declare that someone has taught them to access and use the Internet, another 30.18% (463/1534) say that they have never been taught or informed by any adult. In our sample, a gender difference emerges: female children appear to be more protected. Girls receive more advice from parents and tutors, and are more willing to talk about what they do and how they behave on the Internet (30.24%, 464/1534 vs 28.48%, 437/1534 of males). Parental involvement and tutoring may therefore be strengthened in order to allow teens to better understand and manage their behavior on the Internet as well as be more aware of the risks to which they could be exposed. Nearly, 61.08% (937/1534) of our teens declared not to have told anyone what they have seen or done on the Internet. This is a point that should be further explored in order to disentangle the possible variables that could lead to such behavior and assess appropriate means of risk prevention.

One of the most intriguing possibilities of using social networking platforms (such as Facebook, Twitter, and so on) is amplifying and changing the social support network of the children. This becomes possible because new technologies lighten the cognitive and mnemonic load normally required for the construction and maintenance of social relationships, and also because they are able to overcome the limitations of time and space typical of more traditional forms of interaction. It is interesting to see if this really happens and, if so, to which extent. According to the social brain hypothesis [37-40] developed by anthropologist and evolutionary psychologist Robin Dunbar, the high degree of encephalization reached by the neocortex of social mammals, especially by Homo sapiens, may be a direct consequence of the cognitive demands required for the stable management of complex relationships that occur within social groups in response to different environmental pressures. Through statistical analysis and comparisons with other animal taxa and other kinds of primates, Dunbar has come to identify an approximate number of 150 friends or social relationships that the individual human subject is actually able to maintain.

The new generation of social platforms like MySpace, Facebook, and Twitter, however, seem to refute this conclusion since coming across profiles with more than 500 or 1000 “friends” is now the norm. Of the students who completed our questionnaire, 28.55% (438/1534) reported having 200-500 friends on Facebook, 20.92% (321/1534) more than 500 friends, whereas 16.42% (252/1534) reported having over 1000 friends. Dunbar analyzed this issue in a recent article [41,42], and according to him, the circle of friends on the social media is not qualitatively homogeneous as it tends to include a vast array of simple acquaintances that people do not foster in a stable (and therefore cognitively demanding) way. The Internet can bypass the biological and cognitive constraints imposed by stable social relationships and offer a new way to better maintain the already existing social networks of the subject, as well as allowing an extension to the layer of weaker, unstable, or indirect connections.

We are witnessing a paradigm shift in regards to the evaluation of social consequences when it comes to new digital technologies and their impact on the lives of adolescents [43]. Although in the 1990s an overall “pessimistic” conception supported by numerous studies [44] prevailed, the current picture appears to be more optimistic [21,2,8,42,9]. Today, both the portion of the population that has access to the Internet and the overall frequency of access have definitely increased. Although some years ago maintaining Web-based contact with people known in real life was very difficult as most of them did not use it, the situation has changed radically. This is confirmed by the results of our survey: 40.09% (615/1534) of respondents personally know all the contacts on their Facebook profile (only 2.93%, 45/1534 declare not to know anyone), and 72% (1094/1534) of the subjects use their own real name and surname on social networks. The principal reason for this change resides is that online communication in the 1990s was mainly based on blogs and chat rooms, and it used to occur among strangers with fictitious names. The new social platforms (ie, Facebook) and instant messaging services on cell phones tend instead to be closer to a non-physical place of meeting between real friends and acquaintances, to which are added layers of weaker and more volatile Web-based connections between strangers. Far from being necessarily bad, these accessory networks could have a positive impact on the psychosocial development of the teen, his or her self-esteem, and sense of social effectiveness [34].

Regarding the potential impact that new digital technologies have on the cognitive development of kids and teens, the debate is very open and the research is still in its early stages. Our teenagers live and grow in a world that increasingly resembles a “game room,” among tools that guarantee access and are able to convey a lot of information at rates that were unimaginable until recently, forcing the receiver to process everything instantly and give quick answers. The frantic revision of information, the speed in processing responses, and switching from one task to another mark the times of a perhaps not harmonious, but faster psychomotor development. The high pervasiveness, accessibility, and frequency of use of these new technologies, now used daily and increasingly to accomplish a variety of tasks (from research and exchange of information, to gaming), appear to offer the possibility of enhancing, at least partially, our cognitive potential. As pointed out by Mark Prensky [45], digital tools (such as online databases, Google’s search engine, computer simulation, and so on) help us overcome our memory constraints, enhancing our daily data gathering, decision-making processing, and access to alternative perspectives.

These observations are in line with some ground-breaking philosophical conceptions of the human mind according to which our cognitive processes are better understood from an externalist, theoretically driven point of view [46-59]. In short, these perspectives reject the classical internalist and neurocentric cognitive paradigm that confines our mental processing merely to the brain, stressing on the contrary the important role that the whole body system along with its constant interaction with both environmental and sociocultural factors play. Apart from the discussion concerning the practical criteria identified by proponents of this approach to delimit the range of environmental factors that could be considered genuine extensions of our cognitive processes [46,47,49-52], this new theoretical paradigm seems to be particularly well suited to achieving a better conceptual grasp of the massive role that the Internet and new digital technologies have come to play in our routine daily life [53-59]. It should, therefore, be further explored experimentally.

Understandably, these changes have triggered concerns related to the fact that such devices could eventually impoverish our sensory-motor, mnemonic, and cognitive skills, because of their partial integration in some external storage devices and the lack of focus associated with multitasking behaviors [60,61]. However, it seems that this kind of pessimistic conclusions suffer from the classic “fear of the novel” associated with the emergence of new technologies. They also ignore the fact that the Internet is certainly not the first technological innovation that has been able to change our behavior and our cognitive functions in some way.

As pointed out by Loh and Kanai in 2016 [61], “throughout our evolutionary history, our cognitive system had been reshaped by the advent of tool making and usage, language, writing, and arithmetic systems.” These kinds of technological changes cannot be stopped and are not necessarily detrimental. As an instance, according to recent behavioral and neurophysiological studies, there is evidence that videogame training can induce neuroplastic changes associated with improvement of executive function, working memory, top-down attention, and visuospatial processing [62-65]. In light of these considerations, categorizing these changes as negative or positive per se seems naive, and future studies should focus on the analysis of causal relationships between the different variables involved in the many uses of such devices.

### Conclusions and Limitations

Our survey provides an interesting snapshot of the frequency and methods of Internet usage of a highly representative sample of Italian adolescents allowing us to obtain also important information on possible risks and dangers, and the level of awareness of teens on the Internet. The extent and characteristics of the phenomena (the Internet usage and the behaviors of teenagers while using the Internet) have not been scientifically studied yet, leading to both a poor understanding and a general concern regarding its potentially dangerous social consequences. Building on these considerations, our study is essentially descriptive, and therefore, it mainly intends to contribute to a deeper understanding of the phenomenon in some of its many facets. The kind of information we were able to collect represents for these reasons a valuable starting point that could help in overcoming the huge generational gap which now exists between teens and their mentor figures due to the incredibly fast development of new digital technologies. This data allows us to assess many outcomes and risk factors to: (1) better comprehend the social consequences of the phenomenon; and (2) generate hypotheses about potential strategies of intervention. However, it cannot isolate causal relations between variables, so future experimental designs and longitudinal studies are required for the further development of the field. Another important goal we have pursued is to provide an effective instrument of investigation for future studies on the subject.

However, some limitations have to be taken into account. First of all, these kinds of technologies are in constant development, so we must be well aware that the situation described here may no longer have the same validity in a few years. We also need to keep in mind that the frequency and pattern of use of these technologies are strongly influenced by conditions of technological and cultural development of the population object of the study, and therefore, the results cannot have universal validity and some differences could be observed even at the regional level within the same country. However, overall this study gives us an accurate picture of the main features of this new reality in 2014, and the high representativity of the sample allows a good generalizability of data results.

We chose to use self-report surveys with close ended multiple questions due to technical reasons (large sample and data analysis). We are conscious that this format could be susceptible to various cognitive biases related to memory errors, underestimation of the real time spent on the Internet due to multitasking, and desire to appear more popular or hide information perceived as socially compromising. We tried to limit the latter problem by making the questionnaire totally anonymous (see “Methods” section). Finally, the currently available literature on the field rarely tends to consider the different activities associated with Internet usage separately (ie, information search, game, social network maintenance, work, and/or study) in relation to the possible impact that these could have on the psychosocial development of children and teens. This inevitably results in the tendency to draw vague and contradictory conclusions on the whole phenomenon [32]. Yet the use of the Internet cannot be conceived as a monolithic, unitary phenomenon, and its positive and negative effects should be better investigated by evaluating each time the type of activity in which the subject is involved [22]. Therefore, future studies should begin to pay more attention to this aspect in order to achieve a more detailed and specific understanding, rather than level it out into general and consequently unrealistic categories.

## Appendix

Multimedia Appendix 1 Original survey form (in Italian).
