# Supplementary material for: Internet Use and Access, Behavior, Cyberbullying, and Grooming: Results of an Investigative Whole City Survey of Adolescents
Source: Interact J Med Res. 2017 Aug 29;6(2):e9. doi: 10.2196/ijmr.6231 (PMC5596301; doi:10.2196/ijmr.6231)
Supplement: Multimedia Appendix 1 [file ijmr_v6i2e9_app1.pdf]

UMG

Dulcium sapientiae initium

QUESTIONARIO CONOSCITIVO

data del test		quanti anni hai?		sesso	M		F	

01	a casa hai un computer tutto tuo?	SI' ☐	NO ☐
02	il tuo computer è dotato di “antivirus”?	SI' ☐	NO ☐
03	hai un collegamento ad Internet?	SI' ☐	NO ☐
04	la tua connessione è abbastanza veloce?	SI' ☐	NO ☐
05	a che età hai cominciato ad usare Internet?	ANNI ☐	
06	qualcuno ti ha insegnato a navigare in Rete?	SI' ☐	NO ☐
07	chi ti ha guidato ad utilizzare Internet?	GENITORI ☐	
		INSEGNANTI ☐	
		AMICI ☐	
		FAMILIARI ☐	
		NESSUNO ☐	
08	ti connetti online solo da un computer fisso?	SI' ☐	NO ☐
09	hai a disposizione solo per te anche un tablet o uno smarphone?	SI' ☐	NO ☐
10	utilizzi tablet o smartphone per navigare in Rete?	SI' ☐	NO ☐
11	per andare su Internet adoperi computer, tablet o smartphone di altri?	GENITORI ☐	
		AMICI ☐	
		NO ☐	

12	i tuoi genitori navigano su Internet con te?	SPESSO [ ] / OGNI TANTO [ ] / MAI [ ]
13	i tuoi genitori hanno installato dei filtri per limitare la tua navigazione in Rete?	SI' [ ] / NO [ ] / NON SO [ ]
14	credi che l'esperienza pratica di un adulto possa aiutare per non sbagliare sul web?	SI' [ ] NO [ ]
15	genitori, insegnanti o amici ti hanno dato qualche consiglio sull'uso di Internet?	SI' [ ] NO [ ]

se SI', quali?

15-A	mi hanno detto di non diffondere mai i miei dati personali	SI' [ ] NO [ ]
15-B	hanno fissato un tempo massimo per la navigazione	SI' [ ] NO [ ]
15-C	mi hanno detto di evitare di comunicare con gli sconosciuti	SI' [ ] NO [ ]
15-D	mi hanno detto a quali siti mi posso collegare e cosa posso fare	SI' [ ] NO [ ]
15-E	mi hanno detto di non inviare o pubblicare foto mie o della famiglia	SI' [ ] NO [ ]
15-F	mi hanno detto come devo comportami in caso di difficoltà, disagio o paura	SI' [ ] NO [ ]
15-G	mi hanno spiegato come usare le chat e i sistemi di messaggistica	SI' [ ] NO [ ]
15-H	mi hanno raccomandato di non usare Internet da solo	SI' [ ] NO [ ]
15-I	mi hanno consigliato di raccontare a loro quel che combino in Rete	SI' [ ] NO [ ]

16	hai mai raccontato a genitori, insegnanti o amici quel che hai visto o fatto online?	SI' [ ] NO [ ]
17	ogni giorno quanto tempo trascorri collegato ad Internet?	UN'ORA O MENO [ ] / 2 – 3 ORE [ ] / 4 – 6 ORE [ ] / PIU' DI* 6 ORE [ ]

18	per quale motivo ti colleghi ad Internet?

puoi dare anche più di una risposta…

18-A	effettuare ricerche scolastiche	SI'		NO	
18-B	trovare informazioni e novità su attualità, musica, sport…	SI'		NO	
18-C	giocare online	SI'		NO	
18-D	ascoltare/vedere o scaricare musica e video	SI'		NO	
18-E	chattare o scambiare messaggi	SI'		NO	
18-F	scrivere o ricevere mail con la posta elettronica	SI'		NO	

19	sei iscritto a qualche social network?	SI'		NO	

se SI', quali?

19-A	Facebook	SI'		NO	
19-B	Twitter	SI'		NO	
19-C	MySpace	SI'		NO	
19-D	Google+	SI'		NO	
19-E	Pinterest	SI'		NO	
19-F	Flickr	SI'		NO	
19-G	YouTube	SI'		NO	
19-H	Instagram	SI'		NO	
19-I	altri ………………………………………………….	SI'		NO	

20	usi una webcam per collegarti con gli amici attraverso Internet?	SI'		NO	

21	quando adoperi la webcam?

anche qui puoi dare anche più di una risposta…

IN VIDEOCHAT CON AMICI E COMPAGNI DI SCUOLA	
IN VIDEOCHAT CON AMICI CONOSCIUTI IN RETE	
PER CONOSCERE MEGLIO NUOVE PERSONE TROVATE ONLINE	
FARE FOTO E VIDEO DA CONDIVIDERE IN RETE	

QUESTIONARIO CONOSCITIVO

22	quando sei in Rete, adoperi un soprannome, un nickname o una falsa identità?	SI’	NO
23	su Internet preferisci agire con un falso profilo?	SI’	NO
24	quando sei su Facebook o su un altro sito ti senti più forte che nella vita reale?	SI’	NO
25	ti piace raccontare bugie quando sei sui social network?	SI’	NO

26	dove hai più amici?	NELLA VITA REALE	SU FACEBOOK E INTERNET

27	quanti amici hai su Facebook?	MENO DI 50
		TRA 50 E 200
		TRA 200 E 500
		TRA 500 E 1000
		PIU’ DI 1000
28	quanti amici di Facebook conosci davvero?	NESSUNO
		POCHI
		TRA 20 E 50
		TRA 50 E 100
		PIU’ DI 100
		TUTTI

29	hai letto con attenzione le regole o le condizioni d’uso di Facebook?	SI’	NO
30	ti sei mai trovato in una situazione spiacevole mentre eri collegato a Internet?	SI’	NO
31	hai mai usato Internet per vendicarti o per fare un dispetto o una cattiveria a qualcuno?	SI’	NO
32	hai paura che qualcuno possa parlare male di te su Facebook o su Internet?	SI’	NO
33	quando chatti, parli di argomenti che ti vergogneresti a trattare di persona?	SI’	NO

34	hai mai ricevuto inviti ad incontrare di persona gente che hai conosciuto online?	SI'	NO
35	hai mai accettato appuntamenti con sconosciuti che ti hanno contattato online?	SI'	NO
36	pubblichi foto e filmati sulla Rete?	SI'	NO
37	quante foto hai pubblicato sul tuo profilo Facebook?	NESSUNA<br>UNA<br>MENO DI 20<br>TRA 20 E 50<br>PIU' DI 50	
38	hai mai spedito o pubblicato foto o video di cui ti potresti vergognare?	SI'	NO
39	scambieresti una tua foto con una ricarica telefonica o un oggetto "firmato"?	SI'	NO
40	hai mai litigato con qualcuno o insultato un'altra persona online?	SI'	NO
41	ti piacciono immagini e video violenti in Rete?	SI'	NO
42	sai che puoi essere denunciato di certi comportamenti su Internet?	SI'	NO
43	credi che la Polizia potrebbe "beccarti" se combini qualcosa online?	SI'	NO
44	potendo scegliere, cosa preferisci?	un nuovo smarphone	il motorino
45	cosa ti diverte di più?	una giornata in spiaggia	chattare o giocare su Internet
